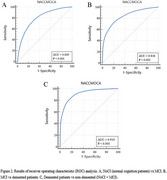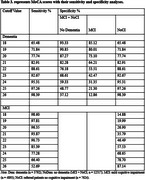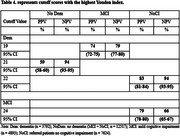# Sensitivity and Specificity of the Montreal Cognitive Assessment: A Retrospective Analysis of 16,309 Participants

**DOI:** 10.1002/alz70857_102635

**Published:** 2025-12-25

**Authors:** Youssef A. Ismail, Huda A. Auf, Shahd A. Sadik, Nada M. Ahmed

**Affiliations:** ^1^ Faculty of Medicine Port Said Univeristy, Egypt, Port Said, Port Said, Egypt

## Abstract

**Background:**

Dementia has been classified as major neurocognitive disorder and it typically presented with substantial impairment in at least one cognitive domain, interfering with day‐to‐day activities and if the impairment was moderate and yet to interfere severely with activities, it would be diagnosed as mild neurocognitive disorder. This study aims to evaluate the sensitivity and specificity of MoCA to determine its suitability as a screening tool in screening programs.

**Methods:**

The study analyzed data from participants aged 55 and older, recruited from U.S. Alzheimer's Disease Research Centers (ADRCs), using a National Alzheimer Coordinating Center Uniformed Data Set (NACC‐UDS). Participants were classified based on patient records into demented and non‐demented groups, with the non‐demented group further categorized into those with normal cognition and cognitive impairment (CI).

**Results:**

The study utilized an initial dataset of 188,700 participant records from NACC. After applying inclusion criteria, 16,309 participants were included. The participants had complete diagnostic information, clinician‐conducted cognitive assessments, and MoCA scores. The participants were categorized into three groups: 7,624 with no cognitive impairment (NoCI), 4,893 with MCI, and 3,792 with dementia. The optimum cutoff scores against NoCI as calculated by the Youden index were less than 24 for detecting MCI, sensitivity 77 % (95% CI, 76‐78), specificity 69 % (95%CI, 67‐69), cutoff scores against MCI as calculated by the Youden index were less than 19 for detecting dementia, sensitivity 72% (95% CI, 70‐73), specificity 80 % (95% CI, 79‐81), and cutoff scores against NoCI as calculated by the Youden index were less than 21 for detecting dementia, sensitivity 83% (95% CI, 82‐85), specificity 82 % (95% CI, 81‐83). Although PPV was generally low, the high NPV across comparisons underscores the MoCA's effectiveness in ruling out cognitive impairment.

**Conclusion:**

The study confirms MoCA as an effective tool for detecting dementia, showing 83% sensitivity and 82% specificity at a cutoff value of 21. With a high NPV of 94%, MoCA is particularly reliable for ruling out dementia. Its ability to identify MCI is moderate, with a sensitivity of 77.3% at cutoff of 24.